# Changes of N6-methyladenosine modulators promote breast cancer progression

**DOI:** 10.1186/s12885-019-5538-z

**Published:** 2019-04-05

**Authors:** Lianpin Wu, Dengying Wu, Jinfeng Ning, Wei Liu, Donghong Zhang

**Affiliations:** 1 Department of Cardiology, The Second Affiliated Hospital of Wenzhou Medical University, 109 Xueyuan Road, Wenzhou, 325027 Zhejiang China; 2 The Thoracic Department of Harbin Medical University Cancer Hospital, 150 Haping Road, Harbin, 150040 China; 3 The Fourth Department of Medical Oncology, Harbin Medical University Cancer Hospital, 150 Haping Road, Harbin, 150040 China; 4 Center for Molecular and Translational Medicine, Georgia State University, 157 Decatur Street SE, Atlanta, GA 30303 USA

**Keywords:** N6-methyladenosine, Breast cancer, Prognosis, Migration; transcription

## Abstract

**Background:**

Breast cancer (BC) displays striking genetic, epigenetic and phenotypic diversity. N^6^-methyladenosine (m6A) in mRNA has emerged as a crucial epitranscriptomic modification that controls cancer self-renewal and cell fate. However, the key enzymes of m6A expression and function in human breast carcinogenesis remain unclear.

**Methods:**

The expression of m6A methylases (METTL3, METTL14 and WTAP) and demethylases (FTO and ALKBH5) were analyzed by using ONCOMINE and The Cancer Genome Atlas databases and in 36 pairs of BC and adjacent non-cancerous tissue. The level of m6A in BC patients was detected by ELISA, and the function of m6A was analyzed by 3-(4,5-dimethylthiazol-2-yl)-2,5-diphenyltetrazolium bromide (MTT) assay, colony formation assay and transwell assay. The database of bc-GenExMiner v4.0, Kaplan-Meier Plotter and cBioPortal were queried for correlation, mutation and prognosis analysis of BC.

**Results:**

The m6A methylases and demethylases were dysregulated in several major malignant tumors. Specifically, the expression of all m6A methylases was reduced in BC as compared with normal controls, but the demethylase ALKBH5 was induced in ONCOMINE databases and confirmed in clinical patients. METTL14 expression was positively correlated with METTL3 expression, and both showed high expression in normal breast-like and luminal-A and -B BC. Functionally, reducing m6A expression by overexpressing METTL14 and/or knockdown of ALKBH5 could inhibit breast cell viability, colony formation and cell migration. Furthermore, Kaplan-Meier, meta-analysis and univariate Cox assay showed that the expression of m6A members including METTL3, METTL14, WTAP and FTO but not their gene mutation and amplification, was tightly associated with cancer progression and poor survival.

**Conclusions:**

Changes of m6A modulators reduced m6A may promote tumorigenesis and predict poor prognosis in BC.

**Electronic supplementary material:**

The online version of this article (10.1186/s12885-019-5538-z) contains supplementary material, which is available to authorized users.

## Background

One of the hallmarks of cancer is dysregulated gene expression. A new concept of the “epitranscriptome” was introduced as a result of transcriptome-wide mapping of N^6^-methyladenosine (m6A), involved in a diverse set of mRNA transcription, splicing, nuclear export, localization, translation, and stability functions [1]. Intense research and accumulating data on the m6A machinery has revealed the major enzymes involved in m6A modification.

The m6A methylation is dynamic, reversible and mediated by multiprotein “writers” (methylases) and “erasers” (demethylases). Writers are methyltransferase-like 3 (METTL3), METTL14 and Wilms tumor 1-associated protein (WTAP). METTL14, together with METTL3, forms a stable heterodimer of methyltransferase complex that mediates cellular m6A deposition on mammalian mRNAs. Knockdown of METTL3 or METTL14 substantially decreases m6A mRNA levels [2]. WTAP itself does not possess methylation activity because it lacks a conserved catalytic methylation domain, but it helps coordinate the localization of the METTL3–METTL14 heterodimer into nuclear speckles, thereby facilitating m6A deposition [3]. The m6A erasers include fat-mass– and obesity-associated protein (FTO) and its homolog AlkB family member 5 (ALKBH5); both selectively reverse m6A to adenosine in nuclear RNA [4]. Thus, identifying the character and regulation of the complex of mammalian m6A methylation machinery is the first step toward deciphering the selectivity and biological functions of m6A deposition in eukaryotic mRNAs.

Although m6A is the most prevalent internal modification that occurs in mRNA, its roles in biological processes have just begun to be uncovered. Previous studies implicated a role for m6A mRNA methylation during carcinogenesis [5], stem cell fate [6], spermatogenesis [4], circadian rhythms [7], and other diseases [8]. Therefore, questions are raised about reversible RNA methylation/demethylation in various malignant tumors. For m6A methylases, METTL3 has been reported to promote the growth and tumorigenesis of human liver cancer cells [9], acute myeloid leukaemia cells [5, 10] and glioblastoma stem cells [11] but also act as a tumor suppressor in renal cell carcinoma [12]. METTL14 expression could also increase the tumorigenesis of glioblastoma stem cells [11] but suppress the metastatic potential of hepatocellular carcinoma [13]. Similarly, WTAP acts as a novel oncogenic protein in acute myeloid leukemia [14], and its expression predicts poor prognosis in malignant glioma [15]. For m6A demethylases, previous studies have suggested that FTO has an oncogenic role in acute myeloid leukemia [16, 17]. Overexpression of ALKBH5 could increase the frequency of the breast cancer (BC) stem-cell phenotype by m6A-mediated demethylation of NANOG gene [18]**.**

However, the roles of distinct writers and erasers of m6A in the tumorigenesis and prognosis of BC, the most common cause of death from cancer in women worldwide, are largely unknown. Accordingly, we explored the expression, association and prognostic effect of m6A enzymes in different clinicopathologic and molecular subtypes as well as the function of m6A in breast tumorigenesis by using data from various databases and verified in clinical patient specimens. We also performed gain- of-function and loss-of-function studies. These findings will be helpful for understanding the pathogenic molecular mechanisms of m6A and for diagnosis.

## Methods

### Patients

We collected samples of fresh BC cancerous tissues and paired normal tissues (at least 5 cm away from the margins) from 36 patients with BC (mean age 49.5 ± 8.7 years) in the Second Affiliated Hospital of Wenzhou Medical University from 2012 to 2015. Tissue histology was confirmed by two pathologists, and patients did not have ductal carcinoma in situ, atypical hyperplasia or benign breast disease. The clinicopathologic parameters of BC were determined according to the World Health Organization histological classification criteria [19].

### Cell culture, transfection and infection

Human MDA-MB-231 cells (Catalog Number: SCSP-5043) were purchased from the Shanghai Institute for Biological Sciences, Chinese Academy of Sciences on the 2/14/2018. MDA-MB-231 cell line was originally derived from pleural effusion of a 51-year-old Caucasian female patient with metastatic breast adenocarcinoma. The identity of the cell line were verified by Single Tandem Repeat (STR) profiling method performed by Eurofins Medigenomix (Ebersberg, Germany) and repeatedly tested in our laboratory for mycoplasma contamination, which could be excluded. We have also double checked the NCBI database for none of misidentification and contamination. The MDA-MB-231 cells were grown in DMEM (Gibco, 11,965) media supplemented with 10% fetal bovine serum (FBS) and 1% 100× Pen Strep (Gibco, 15,140) at 37 °C in a humidified atmosphere containing 5% CO_2_. After culture for 24 h at 70% density, cells were transfected with short-hairpin RNA (shRNA) for ALKBH5 knockdown (Santa Cruz Biotechnology, sc-93,856-SH) and/or pcDNA3/Flag-METTL14 plasmid (Addgene, #53740) for METTL14 overexpression (OE-METTL14) by using Lipofectamine RNAiMAX Reagent or Lipofectamine 2000 Reagent (Thermo Fisher Scientific) following the manufacturer’s protocols. shRNA-A control (Si-CN, Santa Cruz Biotechnology, sc-108,060) and control plasmid (OE-CN) of pcDNA3 Flag HA (Addgene, #10792) were the transfection controls.

### Cell viability assay

Cell viability was measured by Cell Counting Kit-8 (CCK8) assay. Overall, 5000 cells/well of MDA-MB-231 cells were seeded in 96-well plates. After transfection with shRNA or plasmid for 24 h, cells were supplemented with 10 μl CCK8 (Sigma, 96,992) and cultured for 2 h in a 5% CO_2_ and 37 °C incubator. Absorbance was measured at 450 nm wavelength. The effect on cell proliferation was assessed as the percent cell viability, with siRNA- and plasmid-control transfected cells considered 100% viable.

### Colony formation assays

In total, 100 MDA-MB-231 cells transfected with shRNA and/or plasmid were seeded in 6-well ultra-low attachment plates (Corning). The growth medium was replaced every 3 days. Colonies were formed and imaged for colony size calculation at 6 days post-treatment. Colony diameter > 50 μm was counted from three different wells.

### Cell transwell migration assay

MDA-MB-231 cells transfected with shRNA and/or plasmid were incubated at 2 × 10^5^ in transwell inserts with 0.8-μm pore size (Corning, New York, USA) in 24-well plates for 24 h. All cells were suspended in serum-free DMEM and seeded in triplicate in each insert. DMEM supplemented with 10% FBS was used as a chemoattractant. Cells in the bottom inside of the membranes were removed. Migrating cells on the outside membrane were washed and stained with 0.1% crystal violet for 10 min. The migrating cells were calculated by counting 5 randomly chosen fields under a microscope.

### ONCOMINE analysis

The transcriptional levels of m6A enzymes in diverse cancer types and matched normal tissues were determined by analysis of the ONCOMINE database as described [20]. In brief, the analysis was performed online (https://www.oncomine.org) with use of Student *t* test. *P* < 0.05 was considered statistically significant, and the gene rank cutoff was the top 10%.

### BC gene-expression miner v4.0 (BC-GenExMiner v4.0)

bc-GenExMiner v4.0 contains 36 annotated genomic datasets for gene expression, prognostic and correlation. The expression of a target gene was compared by clinical criteria such as age, estrogen receptor status (ER) (by immunohistochemistry [IHC]), progesterone receptor status (PR) (by IHC), HER2 receptor status (HER2) (by IHC), and triple-negative BC and nodal status. Correlation maps and Pearson pairwise correlation plots among m6A family members were used for all patients. Prognostic results are presented by forest plots, univariate Cox analysis and Kaplan-Meier curves.

### Kaplan-Meier plotter

The prognostic merits of candidate genes in human BC were evaluated by using bc-GenExMiner v4.0. The prognostic value was confirmed by using Kaplan-Meier Plotter (www.kmplot.com) [21], with hazard ratios (HRs) and 95% confidence intervals (CIs) and logrank *p* value.

### cBioPortal

The cBioPortal for Cancer Genomics provides both sequencing and pathological data for large-scale cancer genomics datasets [22]. The Molecular TAxonomy of BC International Consortium (METABRIC) [23, 24], a BC dataset containing data for 2509 cases (the largest sample) with pathology reports, was selected for further analysis of m6A enzymes with cBioPortal (www.cbioportal.org) [25] and confirmed with The Cancer Genome Atlas (TCGA) database (1105 samples). Briefly, the genomic profiles included mutations, copy-number variance (CNV) from GISTIC, mRNA expression z-scores (RNA Seq V2 RSEM) and protein expression z-scores (RPPA). The Oncoprint in cBioPortal represents the proportion and distribution of samples with altered m6A enzyme expression. Overall survival for all BC patients was calculated according to the cBioPortal’s online instructions.

### Quantitative RT-PCR (qRT-PCR)

qRT-PCR of gene expression was conducted as we previously described [26, 27]. Briefly, total RNA was isolated and converted to cDNA with use of TRIzol reagent (Life Technologies) and the SuperScript III Reverse Transcriptase kit (Life Technologies). qRT-PCR amplification involved the Power SYBR Green PCR Master Mix (Life Technologies) and Applied Biosystems 7900HT Fast Real-Time PCR System (Invitrogen). Specific primers are in Additional file 1: Table S1. Amplification of β-ACTIN was an internal control. The relative expression of METTL3, METTL14, WTAP, FTO and ALKBH5 in BC tissue was normalized to corresponding normal control tissue. All qRT-PCR analyses were performed in biological triplicates for each sample.

### m6A quantification

The change in global m6A total RNA level was measured by using the EpiQuik m6A RNA Methylation Quantification Kit (Colorimetric) (#P-9005, Epigentek, Farmingdale, NY) according to the manufacturer’s protocol. In total, 200 ng total RNA extracted from clinical BC patients and transfected MDA-MB-231 cells was used for analysis.

### Statistical analysis

All data are representative of at least three independent experiments and results are shown as mean ± SD after the assessment of normality. SPSS 16.0 (SPSS Inc., Chicago, IL) was used for analysis. Student *t* test was used to assess differences in mRNA expression of m6A members between BC and adjacent normal tissue as well as clinicopathological variables. One-way ANOVA followed by Tukey’s test was used for analysis of differences among groups by transfection of shRNA or plasmid. Spearman correlation was used to analyze the correlation between m6A members [28]. *P* < 0.05 was considered statistically significant.

## Results

### Reduced m6A level and methylase expression in BC

**Fig. 1:**
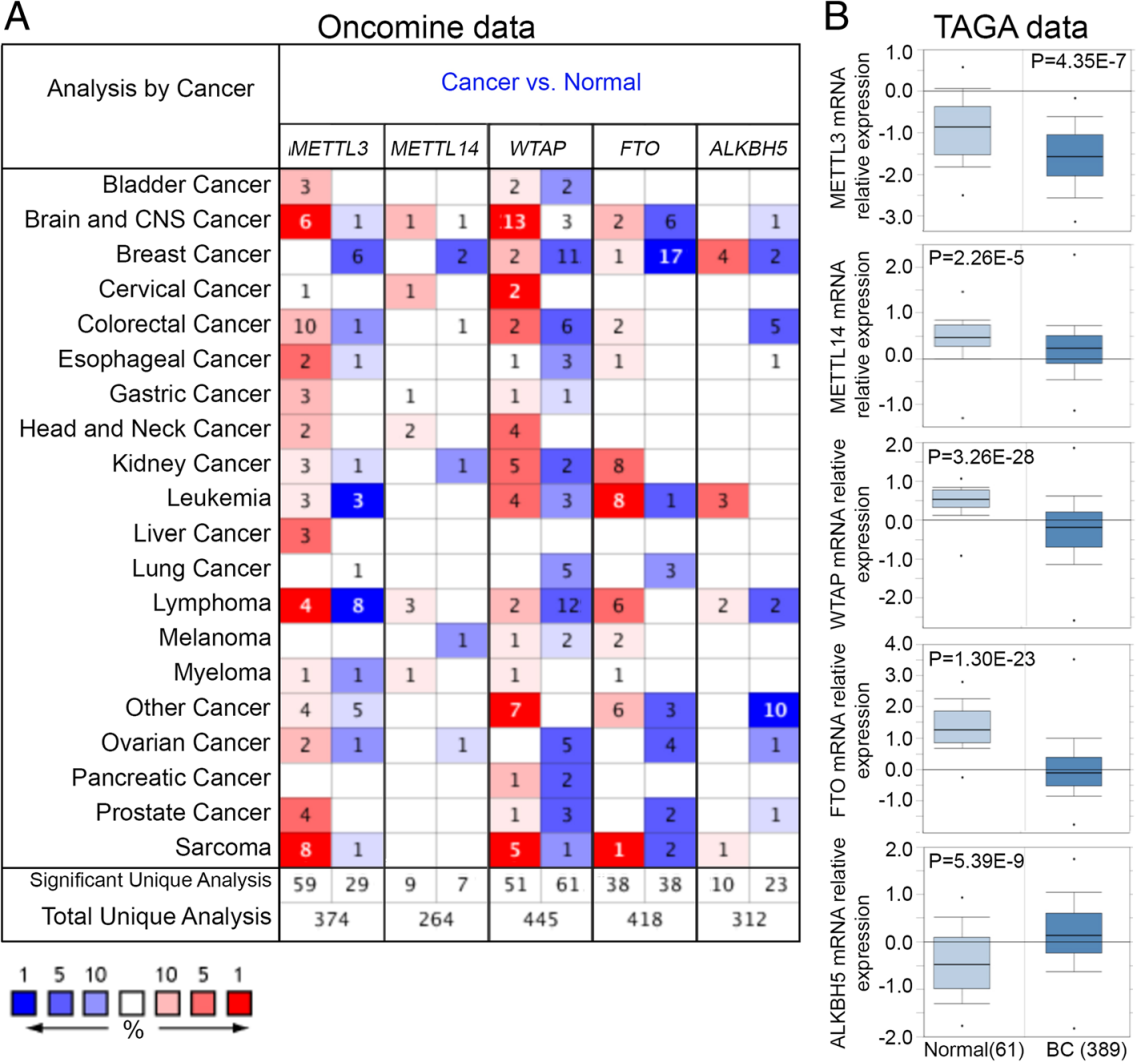
Transcriptional pattern of m6A enzymes in major malignant tumors from ONCOMINE database analysis. **a**, The heat-map represents data with statistically significant upregulation (red) or downregulation (blue) of m6A factors in various human cancer tissues as compared with their normal control. The numbers in the heatmap indicate the published independent datasets of mRNA microarray experiments. **b**, Box plots from gene expression data in the The Cancer Genome Atlas (TCGA) database comparing the expression of specific m6A members, including methylases (METTL3, METTL14 and WTAP) and demetylases (FTO and ALKBH5) in normal and BC tissue

ONCOMINE database analysis revealed that m6A enzymes were distinctively dysregulated in several major malignant tumors. Levels of the m6A methylase “writers,” including METTL3, METTL14 and WTAP, were increased in brain, central nervous system (CNS), cervical, and head and neck cancer and sarcoma but decreased in BC, lymphoma and ovarian cancer as compared with normal controls. Conversely, levels of the m6A demethylase “erasers,” including FTO and ALKBH5, were increased in kidney cancer and leukemia but decreased in BC, brain, CNS and ovarian cancer (Fig. 1a).

**Fig. 2:**
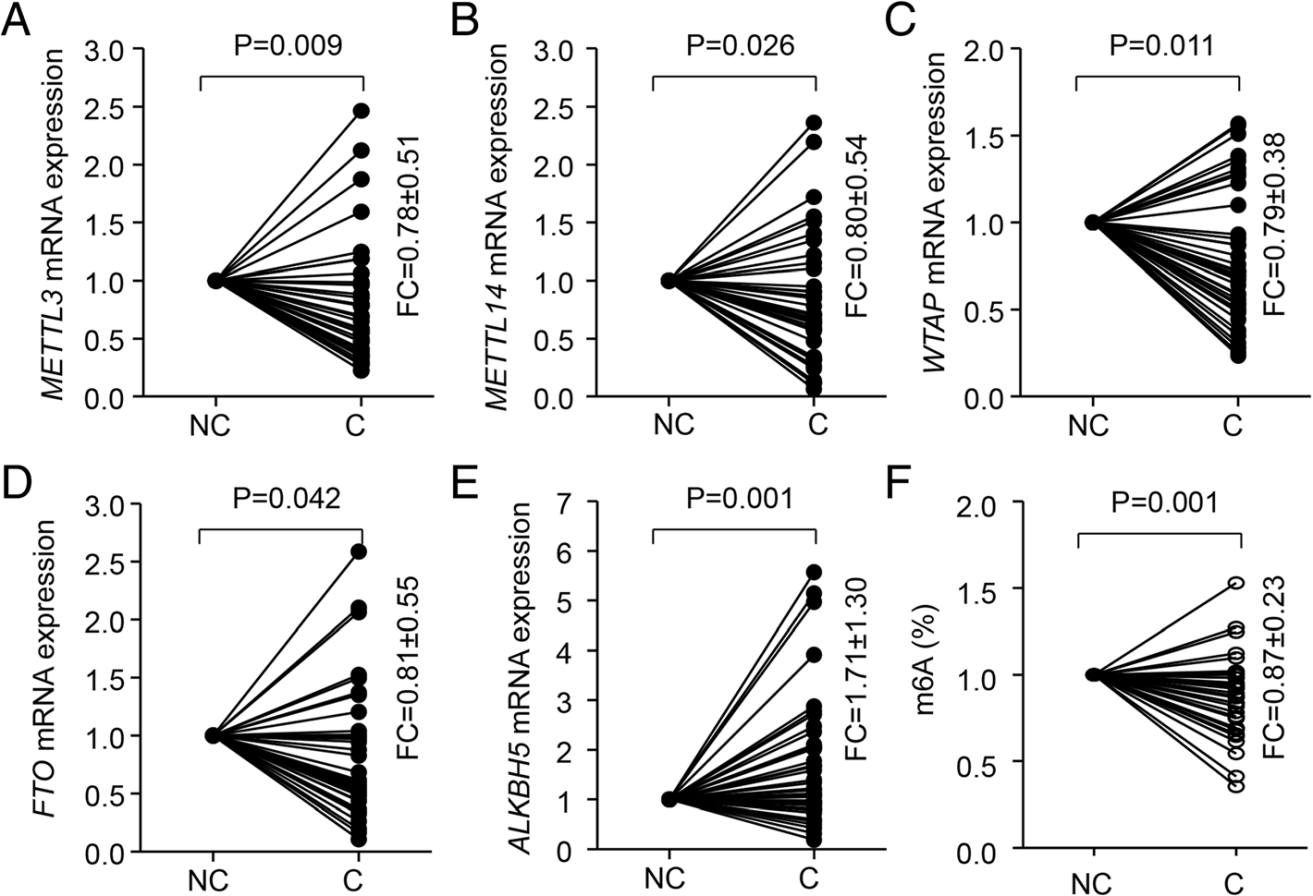
mRNA expression of m6A members in BC patients. qRT-PCR analysis of mRNA expression of m6A members (**a**-**e**) and ELISA analysis globe m6A level (**f**) in breast cancer (**c**) and paired adjacent noncancerous (NC) tissue. FC, fold change

Specifically, the expression of the m6A methylases METTL3, METTL14, and WTAP was reduced 1.575-, 1.200- and 1.883-fold in BC samples as compared with normal tissue, whereas that of the m6A demethylase ALKBH5 was 1.523-fold increased, as derived from TCGA data for BC (Fig. 1b). Consistently, most of these mRNA changes were confirmed by other studies, including Ma et al. [29], Richardon et al. [30], Turashvili et al. [31], Gluck et al. [32], Curtis et al. [24], and Finak et al. [33]. In addition, the above results were verified in our clinical BC patients: with 77.87, 80.19, 69.04 and 81.49% reduction of mRNA expression of METTL3, METTL14, WTAP and FTO and 1.71-fold induction of ALKBH5 mRNA expression in tumor tissue as compared with non-tumor tissue (Fig. 2a-e). Consistently, the decrease in expression of m6A methylases and increase in that of m6A demethylase contributed to reduced m6A level in BC tissue as compared with control tissue (Fig. 2f).

### Increase in m6A inhibited BC growth and metastasis

**Fig. 3:**
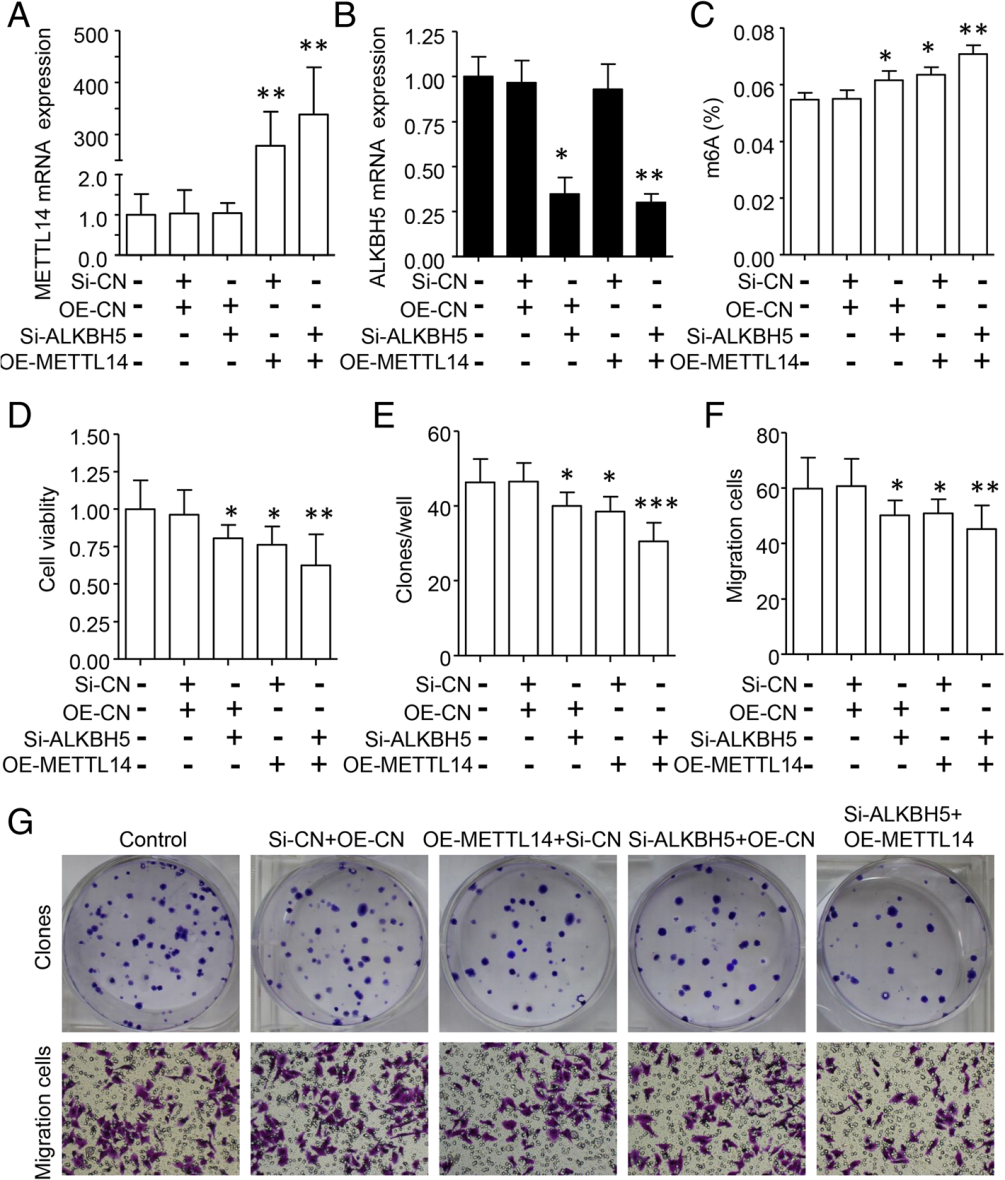
Induction of m6A inhibited BC growth and metastasis. (**a, b**) Stable Overexpression of METTL14 by pcDNA3/Flag-METTL14 and/or shRNA knockdown of ALKBH5 in MDA-MB-231 cells was verified by qRT-PCR assay. Overexpression of METTL14 and/or knockdown of METTL3 significantly induced global m6A level by ELISA assay (**c**) but reduced cell viability by CKK8 assay (**d**), colony formation ability (**e**) and cell migration by transwell assay (**f**). (**g**) Representative images of colony formation and cell migration cells. * *P* < 0.05, ** *P* < 0.01, *** *P* < 0.001

METTL14 and ALKBH5 were reported as oncogene and tumor suppressor genes, respectively [11, 13, 18, 34]. To investigate the functional roles of m6A in BC, we established stable overexpression of METTL14 by transfection with pcDNA3/Flag-METTL14 plasmid and/or knockdown of ALKBH5 by transfection with shRNA in MDA-MB-231 cells (Fig. 3a, b). As expected, overexpression of METTL14 and/or knockdown of ALKBH5 remarkably induced m6A mRNA level (Fig. 3c). Importantly, m6A induction significantly suppressed BC cell viability (Fig. 3d) and inhibited MDA-MB-231 colony-formation abilities (Fig. 3e). Furthermore, m6A greatly suppressed cell migratory abilities (Fig. 3f, g). Thus, increased m6A RNA methylation could inhibit BC growth and metastasis.

### Relationship between m6A enzyme expression and molecular subtype as well as BC clinicopathology

We next used bc-GenExMiner and compared the mRNA expression of m6A enzymes by molecular subtypes of BC. ER(+) or PR(+) status was associated with high mRNA levels of METTL3, METTL14, FOT and ALKBH5 but low level of WTAP as compared with negative status (Table 1). Triple-negative BC (TNBC) is a specific type of BC, with ER(−) and PR(−) status and human epidermal growth factor receptor 2 (HER2)(−) status. We found the same mRNA expression trends in non-TNBC patients. As well, FTO mRNA expression was significantly downregulated in HER2(+) patients, with no significant difference in gene expression by age or nodal status. These observations were confirmed in our clinical patients (Table 2).

**Table 1:** The relationship between m6A enzyme mRNA expression and clinicopathological parameters with breast cancer. (Data from bc-GenExMiner v4.0)

Parameter	Status	METTL3		METTL14		WTAP		KIAA1429		FTO		ALKBH5	
		No.	mRNA	No.	mRNA	No.	mRNA	No.	mRNA	No.	mRNA	No.	mRNA
Age	≤51	1302	1	810	1	1303	1	548	1	1192	1	766	1
	> 51	2051	Nc	1673	Nc	2051	Nc	959	Nc	1896	Nc	1546	Up**
ER (IHC)	–	1488	1	949	1	1376	1	453	1	1383	1	892	1
	+	3810	Up***	2374	Up***	3588	Down***	1282	Up***	3650	Up***	2261	Up**
PR (IHC)	–	871	1	560	1	729	1	270	1	871	1	560	1
	+	1365	Up***	935	Up***	1175	Down***	504	Up***	1365	Up***	935	Up*
HER2 (IHC)	–	1286	1	548	1	1286	1	405	1	1286	1	548	1
	+	175	Nc	134	Nc	175	Nc	122	Down*	175	Down**	134	Nc
TNBC	+	356	1	179	1	356	1	116	1	356	1	179	1
	–	3968	Up***	2494	Up***	3726	Down***	1366	Up**	3808	Up*	2381	Nc
Nodal	–	2375	1	1251	1	2376	1	698	1	2230	1	1251	1
	+	1432	Nc	1064	Up*	1432	Nc	571	Nc	1312	Nc	1064	Nc

*Nc* No, change; *Up* upregulation, *Down* downregualtion, *ER* estrogen receptor, *PR* progesterone-receptor, *HER2* human epidermal growth factor receptor 2, *TNBC* triple-negative breast cancer. ^*^p < 0.05, ^**^p < 0.01, ^***^p < 0.001

**Table 2:** The relationship between m6A enzyme mRNA expression and clinicopathological parameters with clinical patients of breast cancer

Parameter	Status (No.)	METTL3 (mRNA)	METTL14 (mRNA)	WTAP (mRNA)	KIAA1429 (mRNA)	FTO (mRNA)	ALKBH5 (mRNA)
Age	≤51 (16)	0.89 ± 0.54	0.80 ± 0.53	0.80 ± 0.44	2.14 ± 1.31	0.92 ± 1.71	1.59 ± 1.23
	> 51 (20)	0.72 ± 0.50	0.81 ± 0.56	0.79 ± 0.36	1.85 ± 1.24	0.76 ± 0.46	1.78 ± 1.38
ER (IHC)	- (13)	0.55 ± 0.18	0.53 ± 0.30	0.81 ± 0.41	1.44 ± 1.39	0.59 ± 0.63	1.07 ± 0.50
	+(23)	0.89 ± 0.59*	0.93 ± 0.59*	0.78 ± 0.38	2.21 ± 1.12	0.92 ± 0.49*	2.02 ± 1.48*
PR (IHC)	-(15)	0.59 ± 0.23	0.65 ± 0.36	0.78 ± 0.44	1.33 ± 0.90	0.63 ± 0.46	1.08 ± 0.23
	+(21)	0.95 ± 0.63*	0.96 ± 0.64*	0.81 ± 0.35	2.33 ± 1.07*	1.01 ± 0.58*	2.20 ± 1.52*
HER2 (IHC)	-(14)	0.72 ± 0.38	0.81 ± 0.45	0.77 ± 0.36	2.46 ± 1.32	0.74 ± 0.41	1.48 ± 0.71
	+(22)	0.81 ± 0.58	0.81 ± 0.59	0.80 ± 0.40	1.67 ± 1.11	0.86 ± 0.62	1.83 ± 1.55
TNBC	+(4)	0.31 ± 0.07	0.30 ± 0.21	0.60 ± 0.09	0.89 ± 0.36	0.41 ± 0.33	1.13 ± 0.68
	-(32)	0.83 ± 0.51*	0.96 ± 0.54	0.81 ± 0.40	2.08 ± 1.26	0.86 ± 0.56	1.77 ± 1.36

*ER* estrogen receptor, *PR* progesterone-receptor, *HER2* human epidermal growth factor receptor 2, *TNBC* triple-negative breast cancer. ^*^p < 0.05

**Fig. 4:**
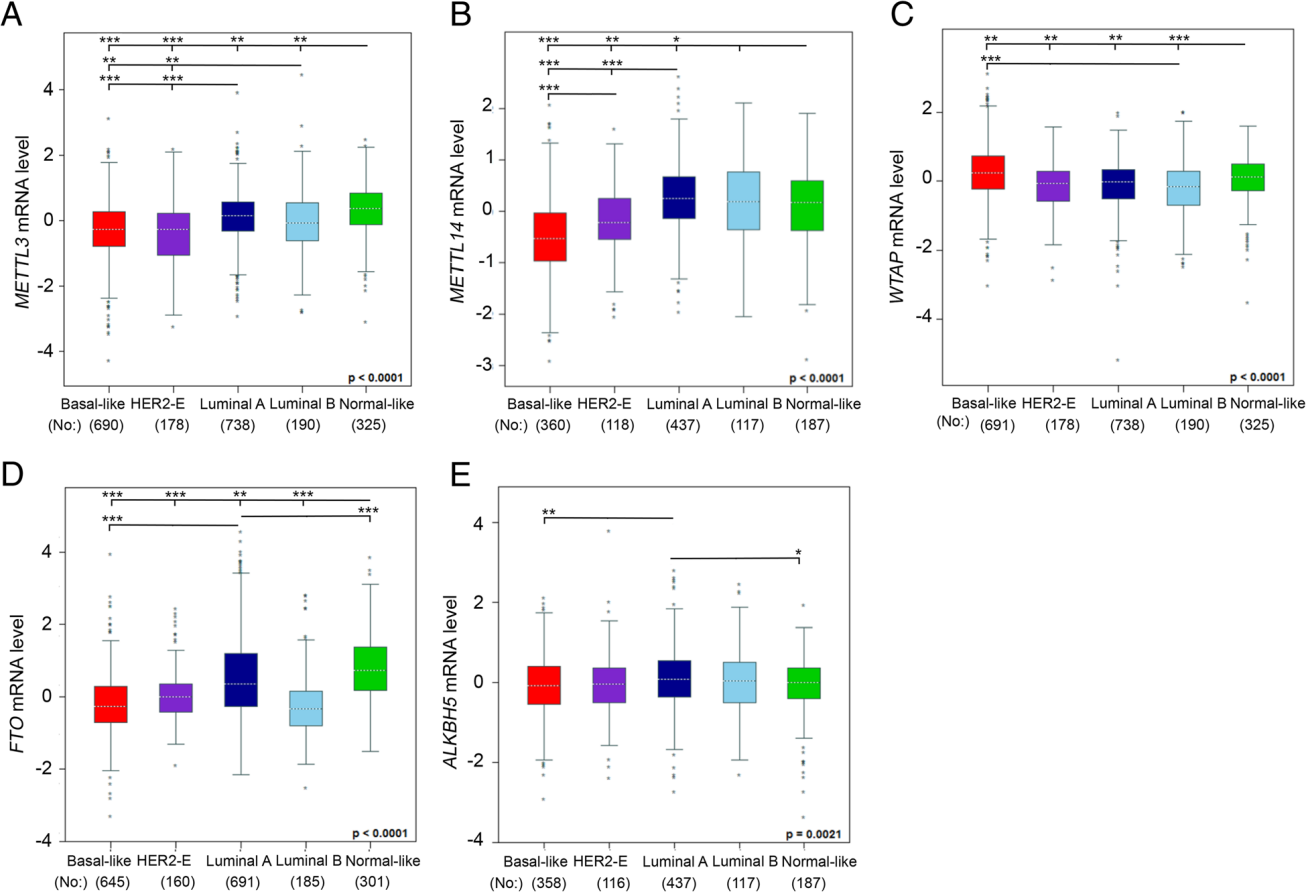
Use of bc-GenExMiner 4.0 database to compare the mRNA expression of m6A enzymes in BC patients by clinicopathologic parameters. (**a-e**) Global significant difference between groups was assessed by Welch’s test, followed by Dunnett-Tukey-Kramer test for pairwise comparison. * *P* < 0.05; ** *P* < 0.01; *** P < 0.001

Furthermore, changes in BC patients differed by clinicopathologic parameters (Fig. 4a-e): both METTL3 and METTL14 were upregulated with normal breast-like and luminal-A and -B BC as compared with basal-like and HER2-E types. mRNA level of FTO was high in normal breast-like and luminal-A BC. In contrast, the highest WTAP mRNA was found with basal-like, then normal breast-like BC. ALKBH5 expression did not change by clinicopathologic features.

### Correlation between m6A enzyme expression in BC

We first used bc-GenExMiner v4.0 correlation analysis to examine the relation between m6A mRNA enzyme expression in BC patients (Table 3). Among m6A methylases, METTL14 expression was positively correlated with METTL3 and WTAP expression. Also, among m6A demethylases, the mRNA expression of FTO and ALKBH5 was positively correlated. The expression of m6A methylases was negatively correlated with that of m6A demethylases, such as FTO and METTL3 and ALKBH5 but not METTL3 or FTO. These results were confirmed in our clinical study (Table 4),

**Table 3:** Pairwise correlation analysis of m6A enzyme levels for all BC patients. (Data from bc-GenExMiner v4.0)

Gene correlation	METTL3	METTL14	WTAP	KIAA1429	FTO
METTL14	r = 0.22*** (*N*= 3374)	1	–	–	–
WTAP	r = −0.03 (*N* = 5014)	r = 0.05** (*N* = 3029)	1	–	–
KIAA1429	r = 0.02 (*N* = 1758)	r = 0.19*** (*N* = 1758)	r = 0.04 (*N* = 1758)	1	–
FTO	r = 0.03* (*N* = 5095)	r = 0.07*** (*N* = 3109)	r = 0.02 (*N* = 4749)	r = −0.08** (*N* = 1758)	1
ALKBH5	r = −0.05** (*N* = 3203)	r = 0.15 (*N* = 3204)	r = 0.02 (*N* = 2858)	r = 0.02 (*N* = 1758)	r = 0.04* (*N* = 2938)

*P < 0.05; **P < 0.01; ***P < 0.0001 as determined by Pearson correlation

**Table 4:** Pairwise correlation analysis of m6A enzyme levels for clinical BC patients

Gene correlation	METTL3	METTL14	WTAP	KIAA1429	FTO
METTL14	r = 0.312 (*P* = 0.031)	1	–	–	–
WTAP	r = 0.185 (*P* = 0.101)	r = 0.24 (0.084)	1	–	–
KIAA1429	r = 0.104 (*P* = 0.441)	r = 0.111 (*P* = 0.317)	r = 0.087 (*P* = 0.618)	1	–
FTO	r = 0.41 (*P* = 0.014)	r = 0.134 (*P* = 0.262)	r = 0.041 (*P* = 0.870)	r = −0.11 (*P* = 0.325)	1
ALKBH5	r = −0.355 (*P* = 0.027)	r = −0.371 (*P*= 0.020)	r = 0.14 (*P* = 0.249)	r = 0.08 (*P* = 0.645)	r = 0.301* (*P* = 0.047)

### Reduced expression of m6A enzymes predicts poor prognosis in BC

**Fig. 5:**
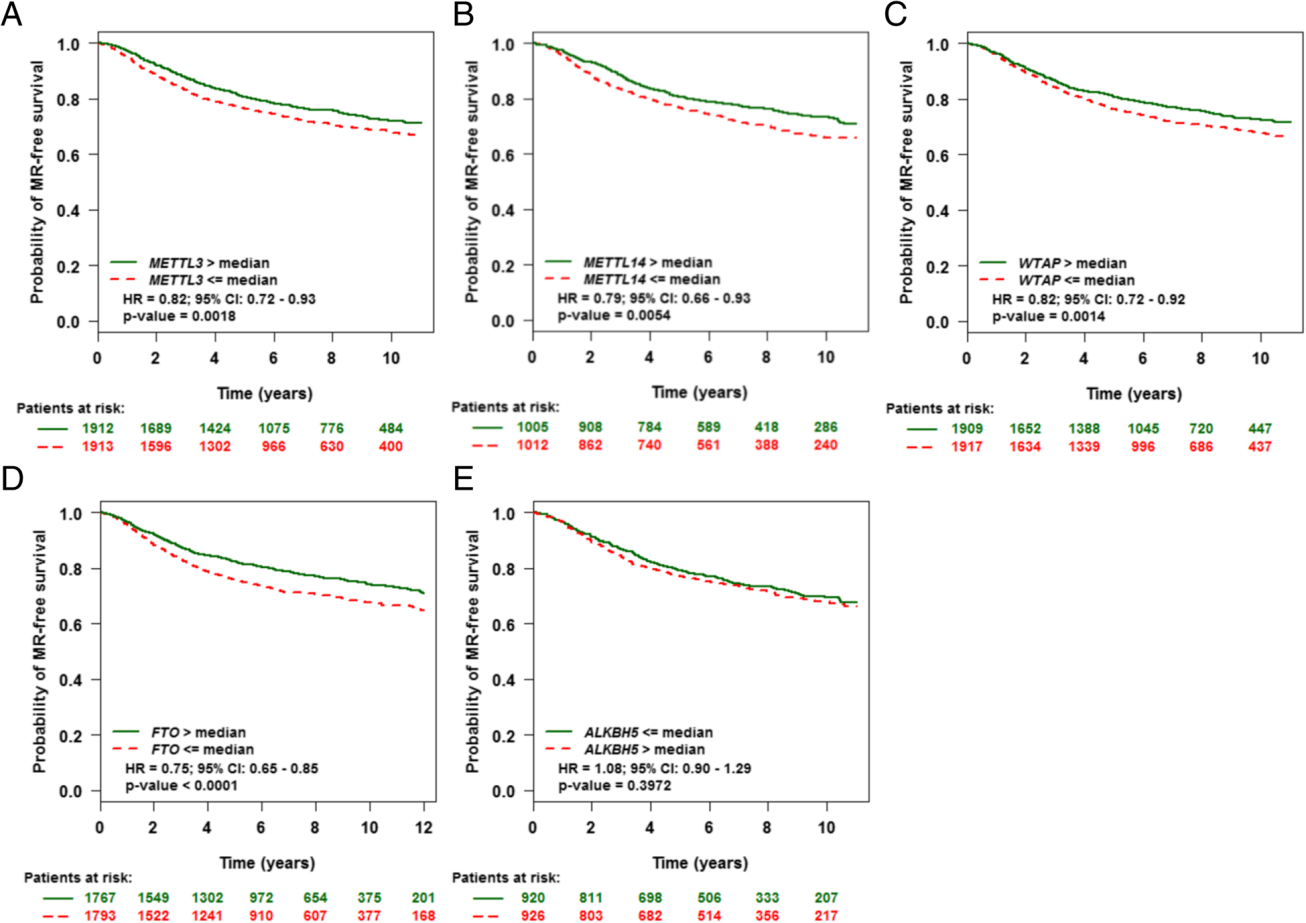
The prognostic value of mRNA level of m6A enzymes in BC (bc-GenExMiner v4.0). Reduced mRNA level of METTL3 (**a**), METTL14 (**b**), WTAP (**c**) and FTO (**d**) but not ALKBH5 (**e**) was significantly associated with poor metastasis relapse (MR)-free survival in all BC patients

Metastasis and biochemical relapse are important factors affecting the survival of BC patients. We next assessed the prognostic value of m6A enzyme levels in patients with BC. Reduced expression of METTL3, METTL14, WTAP and FTO but not ALKBH5 was associated with poor metastasis relapse (MR)-free survival in all BC patients by Kaplan-Meier analysis, meta-analysis (Additional file 1: Figure S1) and univariate Cox analysis (Additional file 1: Table S2-S6) based on the bc-GenExMiner v4.0 database (Fig. 5). Similar prognostic observations were confirmed by using Kaplan-Meier Plotter (Additional file 1: Figure S2).

Univariate analysis indicated both nodal-negative and ER(+) and MR-free survival associated with elevated expression of METTL14 (HR =0.72, *P* = 0.0014), WTAP (HR =0.77, *P* = 0.0001) or FTO (HR =0.74, *P* < 0.0001). A similar pattern was found for METTL3 expression with nodal (−) and ER (−) status and MR-free survival (HR =0.82, *P* = 0.0246). However, ALKBH5 could not predict prognosis for any types of BC (Additional file 1: Tables S7-S11).

### Genetically altered m6A enzymes affect overall but not disease-free survival in BC

**Fig. 6:**
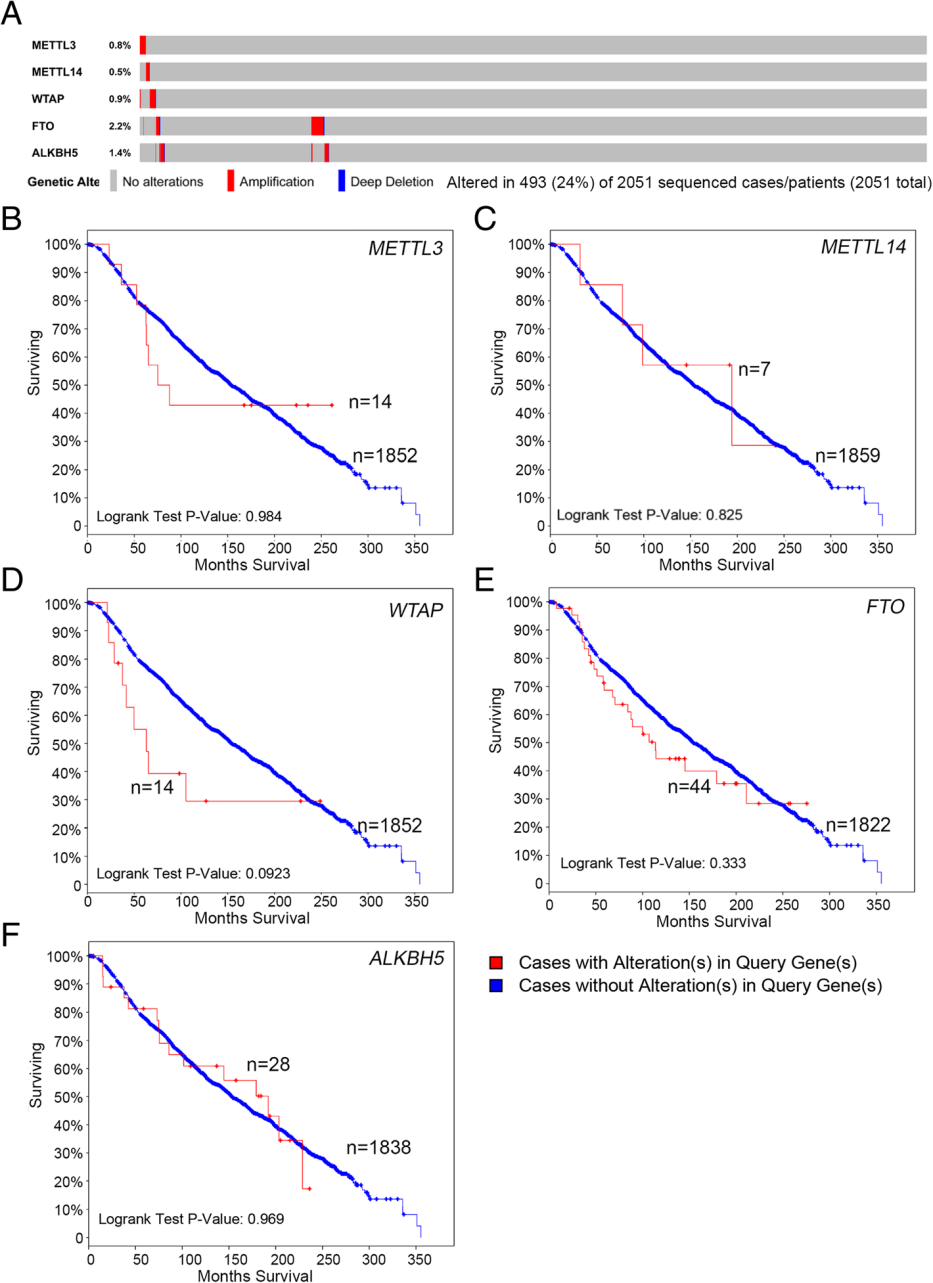
Genetic alteration in m6A enzymes and association with prognosis in breast-invasive carcinoma from cBioPortal data analysis. **a** Oncoprint in cBioPortal was used to represent the proportion and distribution of samples with genetically altered m6A enyzmes in the METABRIC database. **b-f** Kaplan-Meier plots comparing overall survival with and without m6A enzyme alterations

By using cBioPortal assay (METABRIC [23, 24]), we found genetic alterations, including amplification and deletion of m6A enzymes in 493 (24%) samples of 2051 patients with breast-invasive carcinoma (Fig. 6a). However, overall survival did not differ with alterations in all m6A members (METT3, METTL14, WTAP, FTO and ALKBH5), which might be related to the few alterations (< 6% overall) (Fig. 6b-e).

## Discussion

BC is the most common and second leading cause of cancer-related deaths among women worldwide [35]. Because of heterogeneity, the disease exhibits unique somatic mutations and gene dysregulation. Therefore, predicting and monitoring response to treatment and disease progression must be examined longitudinally with next-generation sequencing and array-based technologies, as well as bioinformatics. Recently, the molecule m6A has gained the attention of RNA biologists because it was functionally implicated in various biological processing, including carcinogenesis [36]. Thus, exploring the functional roles and underlying molecular mechanisms of m6A is necessary in BC.

Here, we first determined that the expression of m6A methylases (METTL3, METTL14 and WTAP) was significantly decreased and that of a demethylase (FTO) was increased in BC tissue relative to normal tissue. These patterns were closely correlated with levels of characteristic molecular markers and predicted poor survival in BC. m6A enzymes might serve as novel diagnostic and prognostic indicators of BC. Furthermore, we functionally demonstrated the essential role of m6A in inhibiting BC growth and migration. Therefore, our findings add a new layer of epigenetic alteration that contributes to the progression of BC.

Identifying the expression pattern of m6A methylases and demethylases, which regulate the RNA methylation landscape in the individual cancer patient, is essential for understanding their molecular mechanism, diagnosis and treatment. Similar to previous observations in embryonic stem cells [6], by using multiple online transcriptome data available for various cancers, we found that the contradicting functions of m6A members might be required for specific cancer development. For instance, the increased expression of METTL3 and METTL14 in brain and CNS cancer might explain their promotion role in growth and tumorigenesis of glioblastoma stem cells [11]. These results indicate that upregulation of METTL3 and METTL14 is required for the development of glioblastoma. Similarly, upregulation of FTO is necessary for its oncogenic role in acute myeloid leukemia [16]. Previous studies indicated that ALKBH5 maintains tumorigenicity and proliferation of BC stem cells and glioblastoma stem-like cells [18, 34, 37]. In at least three database searches, including the largest population (593 samples) in the TCGA data, we found a consistent decrease in expression of m6A enzymes during BC development. However, we could not implicate the oncogenic role of METTL3 in BC because the expression of METTLE3 was consistently attenuated in findings from the TCGA and Gluck databases and in several studies [13, 23, 24, 30, 31]. Consistent with a recent study [38], decreased METTL3 expression combined with increased ALKBH5 expression contributed to significantly decreased m6A level in genetically defined immortalized and oncogenically transformed human mammary epithelial cells. Functionally, METTL14 was reported as a tumor suppressor for glioblastoma [11] and hepatocellular carcinoma [13] and ALKBH5 as oncogene for glioblastoma [34] and BC [18]. Therefore, overexpression of METTL14 and/or knockdown of ALKBH5 in MDA-MB-231 cells alone or together could reduce m6A level and further inhibit BC growth and migration.

However, findings for m6A and the role of m6A members in carcinogenesis can seem controversial. For instance, METTL3 is reported as an oncogene for lung adenocarcinoma, glioblastoma and hepatocellular carcinoma [36], whereas we found decreased METTL3 expression in BC. A reasonable explanation for this paradox could be the differences in m6A modification status in various tumors as well as cancer heterogeneity, highlighting the differences in m6A target gene repertoires among cancer cells.

In BC tissues, collaboration between RNA m6A member expression and distribution is close. The expression of the methylases METTL3 and METTL14 was positively correlated and high in normal breast-like and luminal A- and B-type BC. A reverse effect was found with demethylases. Furthermore, methylase activity was negatively related to that of demethylases, such as METTL3 versus ALKBH5. Thus, the exact expression and distribution might be necessary to detect the difference in clinicopathological and molecular subtypes of BC. As well, reduced level of the m6A members METTL3, METTL14, WTAP and FTO but not their mutation and overexpression was tightly associated with cancer progression and poor survival, and these could be developed as novel prognostic markers to predict tumor recurrence. Thus, m6A introduction (methylation) and m6A removal (demethylation) may be associated with the malignant phenotype of BC and a promising therapeutic target for clinical BC. Uncovering the crucial functions of m6A patterns in RNA may lead to new treatment ideas for glioblastoma.

## Conclusions

Our study indicated that abnormal mRNA expression but not gene mutation or amplification of m6A enzymes, especially METTL3, METTL14, WTAP and FTO, might be a novel diagnostic and prognostic strategy for BC and contribute to BC development. Determining whether the m6A target gene and pathway in the tumorigenesis of BC and the molecular mechanism is challenging but would be rewarding for illustration in vitro and in vivo. The present study opens a new era for epi-transcriptome research in BC based on m6A function and modification.

## Additional file

Additional file 1: **Figure S1.** Forest plot of mRNA expression of m6A enzymes for metastasis relapse (MR)-free survival in all BC patients by meta-analysis according to the bc-GenExMiner v4.0 database. **Figure S2.** The prognostic value of mRNA level of m6A enzymes in BC patients (RFS in Kaplan-Meier plotter). **Table S1.** List of primers used in qRT-PCR assays. **Table S2.** Univariate Cox analysis of METTL3 for clinical survival of breast cancer patients (data from bc-GenExMiner v4.0). **Table S3.** Univariate Cox analysis of METTL14 for clinical survival of breast cancer patients (data from bc-GenExMiner v4.0). **Table S4.** Univariate Cox analysis of WTAP for clinical survival of breast cancer patients (data from bc-GenExMiner v4.0). **Table S5.** Univariate Cox analysis of FTO for clinical survival of breast cancer patients (data from bc-GenExMiner v4.0). **Table S6.** Univariate Cox analysis of ALKBH5 for clinical survival of breast cancer patients (data from bc-GenExMiner v4.0). **Table S7.** Univariate Cox analysis of the prognostic value of METTL3 in breast cancer by clinicopathological factors. **Table S8.** Univariate Cox analysis of the prognostic value of METTL14 in breast cancer by clinicopathological factors. **Table S9.** Univariate Cox analysis of the prognostic value of WTAP in breast cancer by clinicopathological factors. **Table S10.** Univariate Cox analysis of the prognostic value of FTO in breast cancer by clinicopathological factors. **Table S11.** Univariate Cox analysis of the prognostic value of ALKBH5 in breast cancer by clinicopathological factors. (DOCX 601 kb)

## Abbreviations

ALKBH5: AlkB family member 5
BC: Breast cancer
FTO: Fat-mass– and obesity-associated protein
m6A: N6-methyladenosine
METTL14: Methyltransferase-like 14
METTL3: Methyltransferase-like 3
WTAP: Wilms tumor 1-associated protein
