# Supplementary material for: Changes of N6-methyladenosine modulators promote breast cancer progression
Source: BMC Cancer. 2019 Apr 5;19:326. doi: 10.1186/s12885-019-5538-z (PMC6451293; doi:10.1186/s12885-019-5538-z)
Supplement: Supplementary file 1 — Figure S1. Forest plot of mRNA expression of m6A enzymes for metastasis relapse (MR)-free survival in all BC patients by meta-analysis according to the bc-GenExMiner v4.0 database. Figure S2. The prognostic value of mRNA level of m6A enzymes in BC patients (RFS in Kaplan-Meier plotter). Table S1. List of primers used in qRT-PCR assays. Table S2. Univariate Cox analysis of METTL3 for clinical survival of breast cancer patients (data from bc-GenExMiner v4.0). Table S3. Univariate Cox analysis of METTL14 for clinical survival of breast cancer patients (data from bc-GenExMiner v4.0). Table S4. Univariate Cox analysis of WTAP for clinical survival of breast cancer patients (data from bc-GenExMiner v4.0). Table S5. Univariate Cox analysis of FTO for clinical survival of breast cancer patients (data from bc-GenExMiner v4.0). Table S6. Univariate Cox analysis of ALKBH5 for clinical survival of breast cancer patients (data from bc-GenExMiner v4.0). Table S7. Univariate Cox analysis of the prognostic value of METTL3 in breast cancer by clinicopathological factors. Table S8. Univariate Cox analysis of the prognostic value of METTL14 in breast cancer by clinicopathological factors. Table S9. Univariate Cox analysis of the prognostic value of WTAP in breast cancer by clinicopathological factors. Table S10. Univariate Cox analysis of the prognostic value of FTO in breast cancer by clinicopathological factors. Table S11. Univariate Cox analysis of the prognostic value of ALKBH5 in breast cancer by clinicopathological factors. (DOCX 601 kb) [file 12885_2019_5538_MOESM1_ESM.docx]

Supplement figures:


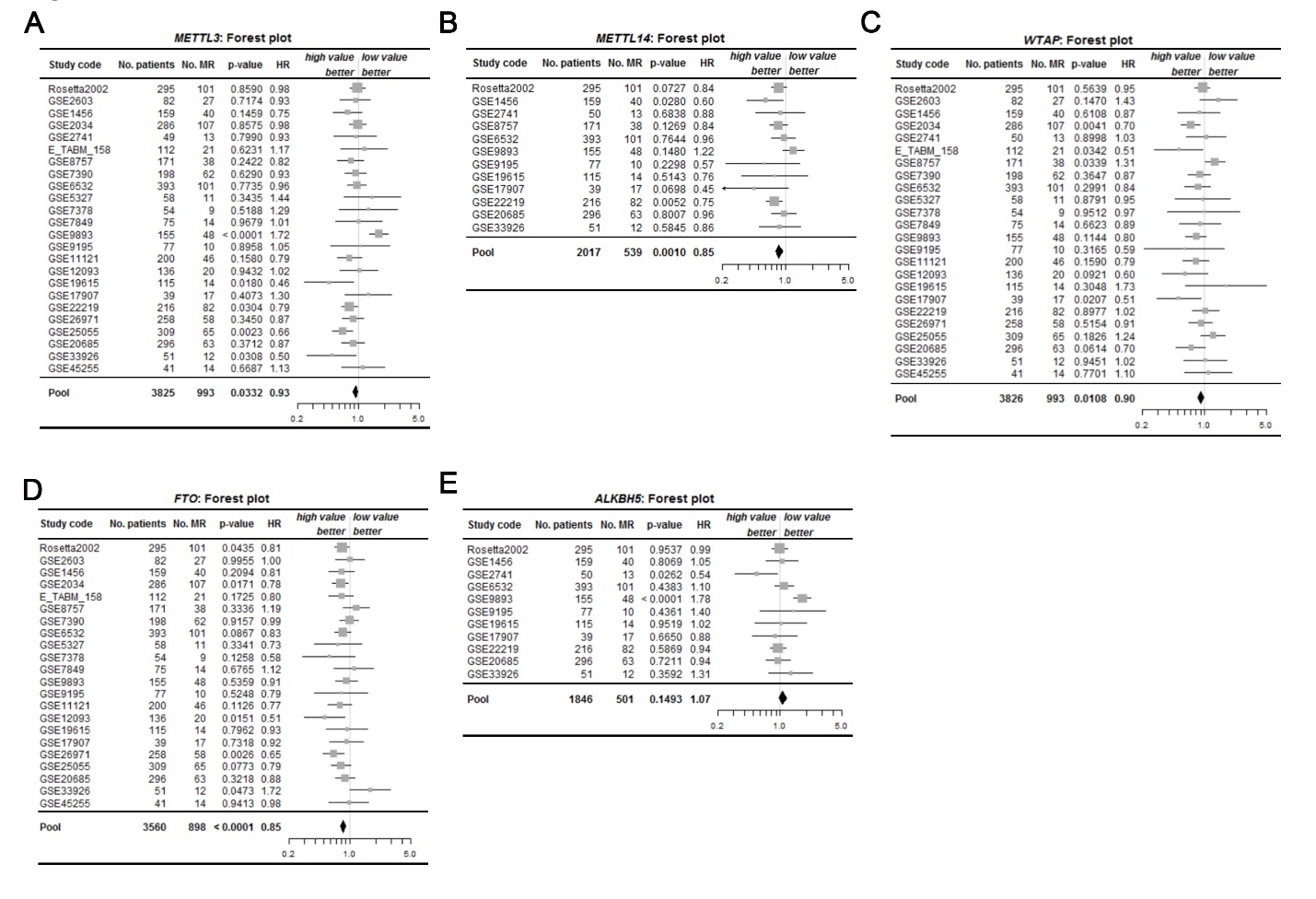


### Figure S1: Forest plot of mRNA expression of m6A enzymes for metastasis relapse (MR)-free survival in all BC patients by meta-analysis according bc-GenExMiner v4.0 database.


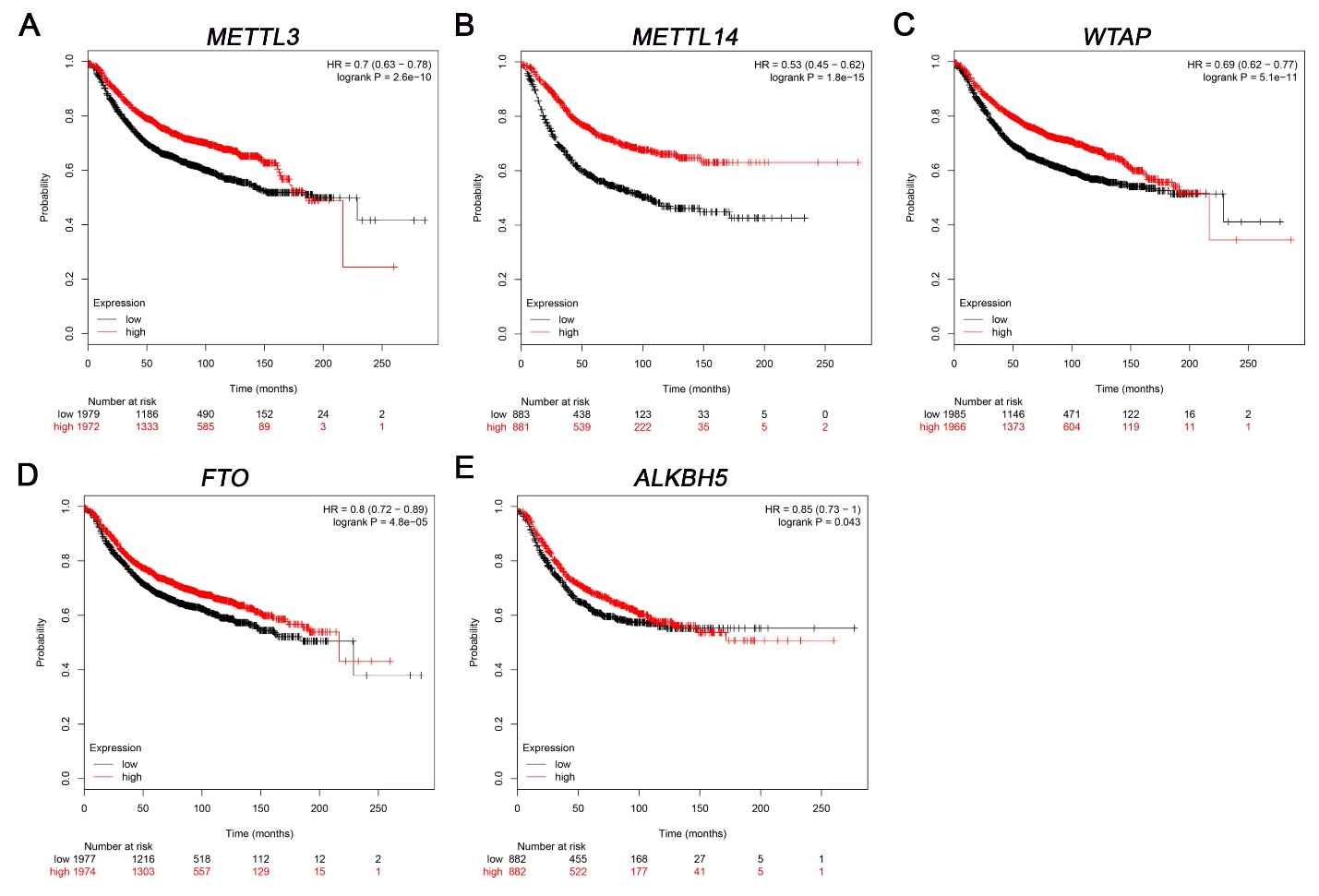


**Figure S2:** The prognostic value of mRNA level of m6A-enzymes in BC patients (RFS in Kaplan-Meier plotter). Reduced METTL3 (A), METTL14(B), WTAP(C), FTO (E) and ALKBH5 (F) but not KIAA1429 (D) and mRNA level was significantly associated with poor overall survival (OS) in all BC patients.

Supplement tables:

Table S1. List of primers used in qRT-PCR assays.

| Assay | Primer sequence (sense 5’-3’) | Primer sequence (anti-sense 5’-3’) | Size (bp) |
| --- | --- | --- | --- |
| METTL3 | TTGTCTCCAACCTTCCGTAGT | CCAGATCAGAGAGGTGGTGTAG | 145 |
| *METTL14* | AGTGCCGACAGCATTGGTG | GGAGCAGAGGTATCATAGGAAGC | 101 |
| *WTAP* | TTGTAATGCGACTAGCAACCAA | GCTGGGTCTACCATTGTTGATCT | 121 |
| *KIAA1429* | TACTTTGAGCCCATTTCTCCTGA | GGAATACTGTCTACTGTTCGTCG | 176 |
| *FTO* | ACTTGGCTCCCTTATCTGACC | TGTGCAGTGTGAGAAAGGCTT | 145 |
| *ALKBH5* | CGGCGAAGGCTACACTTACG | CCACCAGCTTTTGGATCACCA | 128 |
| *β-ACTIN* | TTAGTTGCGTTACACCCTTTC | GCTGTCACCTTCACCGTTC | 156 |

Table S2. Univariate Cox analysis of METTL3 for clinical survival of breast cancer patients. (Data from bc-GenExMiner v4.0).

| Cohort | References | No. patients | No. MR | HR | 95% CI | p-value |
| --- | --- | --- | --- | --- | --- | --- |
| Rosetta2002 | [Van de Vijver et al., 2002](http://www.ncbi.nlm.nih.gov/pubmed/12490681) | 295 | 101 | 0.98 | 0.81–1.20 | 0.859 |
| GSE2603 | [Minn et al., 2005](http://www.ncbi.nlm.nih.gov/pubmed/16049480) | 82 | 27 | 0.93 | 0.62–1.39 | 0.7174 |
| GSE1456 | [Pawitan et al., 2005](http://www.ncbi.nlm.nih.gov/pubmed/16280042) | 159 | 40 | 0.75 | 0.51–1.11 | 0.1459 |
| GSE2034 | [Wang et al., 2005](http://www.ncbi.nlm.nih.gov/pubmed/15721472) | 286 | 107 | 0.98 | 0.78–1.23 | 0.8575 |
| GSE2741 | [Weigelt et al., 2005](http://www.ncbi.nlm.nih.gov/pubmed/16230372) | 49 | 13 | 0.93 | 0.53–1.62 | 0.799 |
| E_TABM_158 | [Chin et al., 2006](http://www.ncbi.nlm.nih.gov/pubmed/17157792) | 112 | 21 | 1.17 | 0.63–2.14 | 0.6231 |
| GSE8757 | [Chin et al., 2007](http://www.ncbi.nlm.nih.gov/pubmed/17925008) | 171 | 38 | 0.82 | 0.58–1.15 | 0.2422 |
| GSE7390 | [Desmedt et al., 2007](http://www.ncbi.nlm.nih.gov/pubmed/17545524) | 198 | 62 | 0.93 | 0.70–1.24 | 0.629 |
| GSE6532 | [Loi et al., 2007](http://www.ncbi.nlm.nih.gov/pubmed/17401012) | 393 | 101 | 0.96 | 0.75–1.24 | 0.7735 |
| GSE5327 | [Minn et al., 2007](http://www.ncbi.nlm.nih.gov/pubmed/17420468) | 58 | 11 | 1.44 | 0.68–3.04 | 0.3435 |
| GSE7378 | [Zhou et al., 2007](http://www.ncbi.nlm.nih.gov/pubmed/17407600) | 54 | 9 | 1.29 | 0.60–2.78 | 0.5188 |
| GSE7849 | [Anders et al., 2008](http://www.ncbi.nlm.nih.gov/pubmed/18167534) | 75 | 14 | 1.01 | 0.55–1.86 | 0.9679 |
| GSE9893 | [Chanrion et al., 2008](http://www.ncbi.nlm.nih.gov/pubmed/18347175) | 155 | 48 | 1.72 | 1.35–2.18 | < 0.0001 |
| GSE9195 | [Loi et al., 2008](http://www.ncbi.nlm.nih.gov/pubmed/18498629) | 77 | 10 | 1.05 | 0.48–2.31 | 0.8958 |
| GSE11121 | [Schmidt et al., 2008](http://www.ncbi.nlm.nih.gov/pubmed/18593943) | 200 | 46 | 0.79 | 0.57–1.09 | 0.158 |
| GSE12093 | [Zhang et al., 2009](http://www.ncbi.nlm.nih.gov/pubmed/18821012) | 136 | 20 | 1.02 | 0.61–1.70 | 0.9432 |
| GSE19615 | [Li et al., 2010](http://www.ncbi.nlm.nih.gov/pubmed/20098429) | 115 | 14 | 0.46 | 0.24–0.88 | 0.018 |
| GSE17907 | [Sircoulomb et al., 2010](http://www.ncbi.nlm.nih.gov/pubmed/20932292) | 39 | 17 | 1.3 | 0.70–2.39 | 0.4073 |
| GSE22219 | [Buffa et al., 2011](http://www.ncbi.nlm.nih.gov/pubmed/21737487) | 216 | 82 | 0.79 | 0.64–0.98 | 0.0304 |
| GSE26971 | [Filipits et al., 2011](http://www.ncbi.nlm.nih.gov/pubmed/21807638) | 258 | 58 | 0.87 | 0.66–1.16 | 0.345 |
| GSE25055 | [Hatzis et al., 2011](http://www.ncbi.nlm.nih.gov/pubmed/21558518) | 309 | 65 | 0.66 | 0.50–0.86 | 0.0023 |
| GSE20685 | [Kao et al., 2011](http://www.ncbi.nlm.nih.gov/pubmed/21501481) | 296 | 63 | 0.87 | 0.65–1.18 | 0.3712 |
| GSE33926 | [Kuo et al., 2012](http://www.ncbi.nlm.nih.gov/pubmed/23049873) | 51 | 12 | 0.5 | 0.26–0.94 | 0.0308 |
| GSE45255 | [Nagalla et al., 2013](http://www.ncbi.nlm.nih.gov/pubmed/23618380) | 41 | 14 | 1.13 | 0.64–2.02 | 0.6687 |
|  | [**Pool**](http://bcgenex.centregauducheau.fr/BC-GEM/GEM-Aide_Popup.php?Aide=Pool) | **3825** | **993** | **0.93** | **0.86–0.99** | **0.0332** |

HR: hazard ratio; 95% CI: 95% confidence interval

Table S3. Univariate Cox analysis of METTL14 for clinical survival of breast cancer patients. (Data from bc-GenExMiner v4.0).

| Cohort | References | No. patients | No. MR | HR | 95% CI | p-value |
| --- | --- | --- | --- | --- | --- | --- |
| Rosetta2002 | [Van de Vijver et al., 2002](http://www.ncbi.nlm.nih.gov/pubmed/12490681) | 295 | 101 | 0.84 | 0.70–1.02 | 0.0727 |
| GSE1456 | [Pawitan et al., 2005](http://www.ncbi.nlm.nih.gov/pubmed/16280042) | 159 | 40 | 0.6 | 0.38–0.95 | 0.028 |
| GSE2741 | [Weigelt et al., 2005](http://www.ncbi.nlm.nih.gov/pubmed/16230372) | 50 | 13 | 0.88 | 0.47–1.65 | 0.6838 |
| GSE8757 | [Chin et al., 2007](http://www.ncbi.nlm.nih.gov/pubmed/17925008) | 171 | 38 | 0.84 | 0.67–1.05 | 0.1269 |
| GSE6532 | [Loi et al., 2007](http://www.ncbi.nlm.nih.gov/pubmed/17401012) | 393 | 101 | 0.96 | 0.71–1.29 | 0.7644 |
| GSE9893 | [Chanrion et al., 2008](http://www.ncbi.nlm.nih.gov/pubmed/18347175) | 155 | 48 | 1.22 | 0.93–1.60 | 0.148 |
| GSE9195 | [Loi et al., 2008](http://www.ncbi.nlm.nih.gov/pubmed/18498629) | 77 | 10 | 0.57 | 0.22–1.43 | 0.2298 |
| GSE19615 | [Li et al., 2010](http://www.ncbi.nlm.nih.gov/pubmed/20098429) | 115 | 14 | 0.76 | 0.33–1.74 | 0.5143 |
| GSE17907 | [Sircoulomb et al., 2010](http://www.ncbi.nlm.nih.gov/pubmed/20932292) | 39 | 17 | 0.45 | 0.19–1.07 | 0.0698 |
| GSE22219 | [Buffa et al., 2011](http://www.ncbi.nlm.nih.gov/pubmed/21737487) | 216 | 82 | 0.75 | 0.61–0.92 | 0.0052 |
| GSE20685 | [Kao et al., 2011](http://www.ncbi.nlm.nih.gov/pubmed/21501481) | 296 | 63 | 0.96 | 0.70–1.31 | 0.8007 |
| GSE33926 | [Kuo et al., 2012](http://www.ncbi.nlm.nih.gov/pubmed/23049873) | 51 | 12 | 0.86 | 0.51–1.47 | 0.5845 |
|  | [**Pool**](http://bcgenex.centregauducheau.fr/BC-GEM/GEM-Aide_Popup.php?Aide=Pool) | **2017** | **539** | **0.85** | **0.78–0.94** | **0.001** |

Table S4. Univariate Cox analysis of WTAP for clinical survival of breast cancer patients. (Data from bc-GenExMiner v4.0).

| Cohort | Reference | No. patients | No. MR | HR | 95% CI | p-value |
| --- | --- | --- | --- | --- | --- | --- |
| Rosetta2002 | [Van de Vijver et al., 2002](http://www.ncbi.nlm.nih.gov/pubmed/12490681) | 295 | 101 | 0.95 | 0.78–1.14 | 0.5639 |
| GSE2603 | [Minn et al., 2005](http://www.ncbi.nlm.nih.gov/pubmed/16049480) | 82 | 27 | 1.43 | 0.88–2.32 | 0.147 |
| GSE1456 | [Pawitan et al., 2005](http://www.ncbi.nlm.nih.gov/pubmed/16280042) | 159 | 40 | 0.87 | 0.50–1.50 | 0.6108 |
| GSE2034 | [Wang et al., 2005](http://www.ncbi.nlm.nih.gov/pubmed/15721472) | 286 | 107 | 0.7 | 0.54–0.89 | 0.0041 |
| GSE2741 | [Weigelt et al., 2005](http://www.ncbi.nlm.nih.gov/pubmed/16230372) | 50 | 13 | 1.03 | 0.62–1.73 | 0.8998 |
| E_TABM_158 | [Chin et al., 2006](http://www.ncbi.nlm.nih.gov/pubmed/17157792) | 112 | 21 | 0.51 | 0.27–0.95 | 0.0342 |
| GSE8757 | [Chin et al., 2007](http://www.ncbi.nlm.nih.gov/pubmed/17925008) | 171 | 38 | 1.31 | 1.02–1.69 | 0.0339 |
| GSE7390 | [Desmedt et al., 2007](http://www.ncbi.nlm.nih.gov/pubmed/17545524) | 198 | 62 | 0.87 | 0.65–1.17 | 0.3647 |
| GSE6532 | [Loi et al., 2007](http://www.ncbi.nlm.nih.gov/pubmed/17401012) | 393 | 101 | 0.84 | 0.60–1.17 | 0.2991 |
| GSE5327 | [Minn et al., 2007](http://www.ncbi.nlm.nih.gov/pubmed/17420468) | 58 | 11 | 0.95 | 0.46–1.93 | 0.8791 |
| GSE7378 | [Zhou et al., 2007](http://www.ncbi.nlm.nih.gov/pubmed/17407600) | 54 | 9 | 0.97 | 0.38–2.50 | 0.9512 |
| GSE7849 | [Anders et al., 2008](http://www.ncbi.nlm.nih.gov/pubmed/18167534) | 75 | 14 | 0.89 | 0.52–1.51 | 0.6623 |
| GSE9893 | [Chanrion et al., 2008](http://www.ncbi.nlm.nih.gov/pubmed/18347175) | 155 | 48 | 0.8 | 0.61–1.05 | 0.1144 |
| GSE9195 | [Loi et al., 2008](http://www.ncbi.nlm.nih.gov/pubmed/18498629) | 77 | 10 | 0.59 | 0.21–1.66 | 0.3165 |
| GSE11121 | [Schmidt et al., 2008](http://www.ncbi.nlm.nih.gov/pubmed/18593943) | 200 | 46 | 0.79 | 0.57–1.10 | 0.159 |
| GSE12093 | [Zhang et al., 2009](http://www.ncbi.nlm.nih.gov/pubmed/18821012) | 136 | 20 | 0.6 | 0.33–1.09 | 0.0921 |
| GSE19615 | [Li et al., 2010](http://www.ncbi.nlm.nih.gov/pubmed/20098429) | 115 | 14 | 1.73 | 0.61–4.95 | 0.3048 |
| GSE17907 | [Sircoulomb et al., 2010](http://www.ncbi.nlm.nih.gov/pubmed/20932292) | 39 | 17 | 0.51 | 0.29–0.90 | 0.0207 |
| GSE22219 | [Buffa et al., 2011](http://www.ncbi.nlm.nih.gov/pubmed/21737487) | 216 | 82 | 1.02 | 0.72–1.46 | 0.8977 |
| GSE26971 | [Filipits et al., 2011](http://www.ncbi.nlm.nih.gov/pubmed/21807638) | 258 | 58 | 0.91 | 0.67–1.22 | 0.5154 |
| GSE25055 | [Hatzis et al., 2011](http://www.ncbi.nlm.nih.gov/pubmed/21558518) | 309 | 65 | 1.24 | 0.91–1.69 | 0.1826 |
| GSE20685 | [Kao et al., 2011](http://www.ncbi.nlm.nih.gov/pubmed/21501481) | 296 | 63 | 0.7 | 0.48–1.02 | 0.0614 |
| GSE33926 | [Kuo et al., 2012](http://www.ncbi.nlm.nih.gov/pubmed/23049873) | 51 | 12 | 1.02 | 0.58–1.78 | 0.9451 |
| GSE45255 | [Nagalla et al., 2013](http://www.ncbi.nlm.nih.gov/pubmed/23618380) | 41 | 14 | 1.1 | 0.59–2.03 | 0.7701 |
|  | [**Pool**](http://bcgenex.centregauducheau.fr/BC-GEM/GEM-Aide_Popup.php?Aide=Pool) | **3826** | **993** | **0.9** | **0.84–0.98** | **0.0108** |

Table S5. Univariate Cox analysis of KIAA1429 for clinical survival of breast cancer patients. (Data from bc-GenExMiner v4.0).

| Cohort | Reference | No. patients | No. MR | HR | 95% CI | p-value |
| --- | --- | --- | --- | --- | --- | --- |
| GSE1456 | [Pawitan et al., 2005](http://www.ncbi.nlm.nih.gov/pubmed/16280042) | 159 | 40 | 0.97 | 0.63–1.49 | 0.8865 |
| GSE6532 | [Loi et al., 2007](http://www.ncbi.nlm.nih.gov/pubmed/17401012) | 393 | 101 | 1.17 | 0.91–1.49 | 0.224 |
| GSE9195 | [Loi et al., 2008](http://www.ncbi.nlm.nih.gov/pubmed/18498629) | 77 | 10 | 0.72 | 0.33–1.56 | 0.3981 |
| GSE19615 | [Li et al., 2010](http://www.ncbi.nlm.nih.gov/pubmed/20098429) | 115 | 14 | 1.46 | 0.68–3.15 | 0.3323 |
| GSE17907 | [Sircoulomb et al., 2010](http://www.ncbi.nlm.nih.gov/pubmed/20932292) | 39 | 17 | 0.77 | 0.43–1.37 | 0.3724 |
| GSE20685 | [Kao et al., 2011](http://www.ncbi.nlm.nih.gov/pubmed/21501481) | 296 | 63 | 1.04 | 0.79–1.37 | 0.7803 |
|  | [**Pool**](http://bcgenex.centregauducheau.fr/BC-GEM/GEM-Aide_Popup.php?Aide=Pool) | **1079** | **245** | **1.05** | **0.90–1.23** | **0.5406** |

Table S6. Univariate Cox analysis of FTO for clinical survival of breast cancer patients. (Data from bc-GenExMiner v4.0).

| Cohort | Reference | No. patients | No. MR | HR | 95% CI | p-value |
| --- | --- | --- | --- | --- | --- | --- |
| Rosetta2002 | [Van de Vijver et al., 2002](http://www.ncbi.nlm.nih.gov/pubmed/12490681) | 295 | 101 | 0.81 | 0.67–0.99 | 0.0435 |
| GSE2603 | [Minn et al., 2005](http://www.ncbi.nlm.nih.gov/pubmed/16049480) | 82 | 27 | 1 | 0.68–1.46 | 0.9955 |
| GSE1456 | [Pawitan et al., 2005](http://www.ncbi.nlm.nih.gov/pubmed/16280042) | 159 | 40 | 0.81 | 0.58–1.13 | 0.2094 |
| GSE2034 | [Wang et al., 2005](http://www.ncbi.nlm.nih.gov/pubmed/15721472) | 286 | 107 | 0.78 | 0.63–0.96 | 0.0171 |
| E_TABM_158 | [Chin et al., 2006](http://www.ncbi.nlm.nih.gov/pubmed/17157792) | 112 | 21 | 0.8 | 0.57–1.11 | 0.1725 |
| GSE8757 | [Chin et al., 2007](http://www.ncbi.nlm.nih.gov/pubmed/17925008) | 171 | 38 | 1.19 | 0.84–1.67 | 0.3336 |
| GSE7390 | [Desmedt et al., 2007](http://www.ncbi.nlm.nih.gov/pubmed/17545524) | 198 | 62 | 0.99 | 0.77–1.26 | 0.9157 |
| GSE6532 | [Loi et al., 2007](http://www.ncbi.nlm.nih.gov/pubmed/17401012) | 393 | 101 | 0.83 | 0.67–1.03 | 0.0867 |
| GSE5327 | [Minn et al., 2007](http://www.ncbi.nlm.nih.gov/pubmed/17420468) | 58 | 11 | 0.73 | 0.39–1.38 | 0.3341 |
| GSE7378 | [Zhou et al., 2007](http://www.ncbi.nlm.nih.gov/pubmed/17407600) | 54 | 9 | 0.58 | 0.29–1.16 | 0.1258 |
| GSE7849 | [Anders et al., 2008](http://www.ncbi.nlm.nih.gov/pubmed/18167534) | 75 | 14 | 1.12 | 0.66–1.89 | 0.6765 |
| GSE9893 | [Chanrion et al., 2008](http://www.ncbi.nlm.nih.gov/pubmed/18347175) | 155 | 48 | 0.91 | 0.68–1.22 | 0.5359 |
| GSE9195 | [Loi et al., 2008](http://www.ncbi.nlm.nih.gov/pubmed/18498629) | 77 | 10 | 0.79 | 0.39–1.62 | 0.5248 |
| GSE11121 | [Schmidt et al., 2008](http://www.ncbi.nlm.nih.gov/pubmed/18593943) | 200 | 46 | 0.77 | 0.56–1.06 | 0.1126 |
| GSE12093 | [Zhang et al., 2009](http://www.ncbi.nlm.nih.gov/pubmed/18821012) | 136 | 20 | 0.51 | 0.30–0.88 | 0.0151 |
| GSE19615 | [Li et al., 2010](http://www.ncbi.nlm.nih.gov/pubmed/20098429) | 115 | 14 | 0.93 | 0.54–1.60 | 0.7962 |
| GSE17907 | [Sircoulomb et al., 2010](http://www.ncbi.nlm.nih.gov/pubmed/20932292) | 39 | 17 | 0.92 | 0.59–1.45 | 0.7318 |
| GSE26971 | [Filipits et al., 2011](http://www.ncbi.nlm.nih.gov/pubmed/21807638) | 258 | 58 | 0.65 | 0.49–0.86 | 0.0026 |
| GSE25055 | [Hatzis et al., 2011](http://www.ncbi.nlm.nih.gov/pubmed/21558518) | 309 | 65 | 0.79 | 0.61–1.03 | 0.0773 |
| GSE20685 | [Kao et al., 2011](http://www.ncbi.nlm.nih.gov/pubmed/21501481) | 296 | 63 | 0.88 | 0.68–1.14 | 0.3218 |
| GSE33926 | [Kuo et al., 2012](http://www.ncbi.nlm.nih.gov/pubmed/23049873) | 51 | 12 | 1.72 | 1.01–2.94 | 0.0473 |
| GSE45255 | [Nagalla et al., 2013](http://www.ncbi.nlm.nih.gov/pubmed/23618380) | 41 | 14 | 0.98 | 0.58–1.65 | 0.9413 |
|  | [**Pool**](http://bcgenex.centregauducheau.fr/BC-GEM/GEM-Aide_Popup.php?Aide=Pool) | **3560** | **898** | **0.85** | **0.79–0.90** | **< 0.0001** |

Table S7. Univariate Cox analysis of ALKBH5 for clinical survival of breast cancer patients. (Data from bc-GenExMiner v4.0).

| Cohort | Reference | No. patients | No. MR | HR | 95% CI | p-value |
| --- | --- | --- | --- | --- | --- | --- |
| Rosetta2002 | [Van de Vijver et al., 2002](http://www.ncbi.nlm.nih.gov/pubmed/12490681) | 295 | 101 | 0.99 | 0.82–1.21 | 0.9537 |
| GSE1456 | [Pawitan et al., 2005](http://www.ncbi.nlm.nih.gov/pubmed/16280042) | 159 | 40 | 1.05 | 0.70–1.59 | 0.8069 |
| GSE2741 | [Weigelt et al., 2005](http://www.ncbi.nlm.nih.gov/pubmed/16230372) | 50 | 13 | 0.54 | 0.31–0.93 | 0.0262 |
| GSE6532 | [Loi et al., 2007](http://www.ncbi.nlm.nih.gov/pubmed/17401012) | 393 | 101 | 1.1 | 0.86–1.41 | 0.4383 |
| GSE9893 | [Chanrion et al., 2008](http://www.ncbi.nlm.nih.gov/pubmed/18347175) | 155 | 48 | 1.78 | 1.42–2.22 | < 0.0001 |
| GSE9195 | [Loi et al., 2008](http://www.ncbi.nlm.nih.gov/pubmed/18498629) | 77 | 10 | 1.4 | 0.60–3.23 | 0.4361 |
| GSE19615 | [Li et al., 2010](http://www.ncbi.nlm.nih.gov/pubmed/20098429) | 115 | 14 | 1.02 | 0.52–1.99 | 0.9519 |
| GSE17907 | [Sircoulomb et al., 2010](http://www.ncbi.nlm.nih.gov/pubmed/20932292) | 39 | 17 | 0.88 | 0.49–1.57 | 0.665 |
| GSE22219 | [Buffa et al., 2011](http://www.ncbi.nlm.nih.gov/pubmed/21737487) | 216 | 82 | 0.94 | 0.77–1.16 | 0.5869 |
| GSE20685 | [Kao et al., 2011](http://www.ncbi.nlm.nih.gov/pubmed/21501481) | 296 | 63 | 0.94 | 0.69–1.29 | 0.7211 |
| GSE33926 | [Kuo et al., 2012](http://www.ncbi.nlm.nih.gov/pubmed/23049873) | 51 | 12 | 1.31 | 0.74–2.33 | 0.3592 |
|  | [**Pool**](http://bcgenex.centregauducheau.fr/BC-GEM/GEM-Aide_Popup.php?Aide=Pool) | **1846** | **501** | **1.07** | **0.97–1.18** | **0.1493** |

Table S8. Univariate Cox analysis of the prognostic value of METTL3 in breast cancer by clinicopathological factors

| Event criteria | | | p value | HR | 95% CI | No. patients | No. events |
| --- | --- | --- | --- | --- | --- | --- | --- |
| N- | ERm | MR | **0.0199** | 0.88 | 0.80–0.98 | 1 886 | 454 |
| N- | ER- | MR | **0.0246** | 0.82 | 0.68–0.97 | 478 | 140 |
| Nm | ER- | MR | **0.029** | 0.88 | 0.78–0.99 | 1 038 | 330 |
| Nm | ERm | MR | **0.0332** | 0.93 | 0.86–0.99 | 3 825 | 993 |
| N- | ERm | AE | 0.1452 | 0.94 | 0.86–1.02 | 2 360 | 719 |
| Nm | ERm | AE | 0.1592 | 0.96 | 0.91–1.01 | 5 291 | 1 804 |
| N+ | ER+ | MR | 0.2169 | 1.1 | 0.95–1.27 | 677 | 202 |
| N- | ER- | AE | 0.3305 | 0.93 | 0.80–1.08 | 611 | 211 |
| Nm | ER- | AE | 0.493 | 0.97 | 0.89–1.06 | 1 476 | 578 |
| N+ | ER+ | AE | 0.6665 | 1.02 | 0.92–1.14 | 983 | 397 |
| N+ | ERm | MR | 0.6862 | 1.02 | 0.91–1.15 | 980 | 322 |
| N+ | ER- | MR | 0.7646 | 0.97 | 0.78–1.20 | 295 | 119 |
| N- | ER+ | MR | 0.7959 | 0.98 | 0.86–1.12 | 1 389 | 312 |
| Nm | ER+ | AE | 0.8628 | 0.99 | 0.93–1.06 | 3 766 | 1 215 |
| N+ | ER- | AE | 0.8687 | 0.99 | 0.84–1.16 | 426 | 210 |
| N+ | ERm | AE | 0.8818 | 1.01 | 0.92–1.10 | 1 418 | 608 |
| Nm | ER+ | MR | 0.9086 | 1.01 | 0.92–1.10 | 2 757 | 658 |
| N- | ER+ | AE | 0.9257 | 0.99 | 0.89–1.11 | 1 724 | 503 |

AE: any event; ER (+,-,m): oestrogen receptor status (+: positive, -: negative, m: mixed); HR: hazard ratio; MR: metastatic relapse; N (+,-,m): nodal status (+: positive, -: negative, m: mixed)

Table S9. Univariate Cox analysis of the prognostic value of METTL14 in breast cancer by clinicopathological factors.

| Event criteria | | | p value | HR | 95% CI | No. patients | No. events |
| --- | --- | --- | --- | --- | --- | --- | --- |
| N- | ERm | MR | **0.0007** | 0.75 | 0.64–0.89 | 762 | 167 |
| Nm | ERm | MR | **0.001** | 0.85 | 0.78–0.94 | 2 017 | 539 |
| N- | ER+ | MR | **0.0014** | 0.72 | 0.59–0.88 | 542 | 113 |
| N+ | ERm | AE | **0.0055** | 0.86 | 0.77–0.96 | 1 050 | 497 |
| N- | ERm | AE | **0.0086** | 0.86 | 0.77–0.96 | 1 236 | 395 |
| N- | ER+ | AE | **0.0096** | 0.82 | 0.71–0.95 | 877 | 276 |
| Nm | ERm | AE | **0.0142** | 0.92 | 0.86–0.98 | 3 325 | 1 250 |
| Nm | ER+ | MR | **0.0327** | 0.88 | 0.78–0.99 | 1 450 | 355 |
| N+ | ER- | AE | 0.0791 | 0.83 | 0.68–1.02 | 266 | 153 |
| N+ | ER- | MR | 0.1275 | 0.79 | 0.58–1.07 | 135 | 68 |
| N+ | ERm | MR | 0.1861 | 0.9 | 0.77–1.05 | 612 | 224 |
| Nm | ER+ | AE | 0.2018 | 0.95 | 0.87–1.03 | 2 349 | 840 |
| Nm | ER- | MR | 0.2928 | 0.91 | 0.77–1.08 | 547 | 181 |
| N- | ER- | MR | 0.2993 | 0.86 | 0.65–1.14 | 205 | 53 |
| N+ | ER+ | AE | 0.4092 | 0.94 | 0.82–1.08 | 781 | 344 |
| N- | ER- | AE | 0.5471 | 0.94 | 0.77–1.15 | 338 | 115 |
| N+ | ER+ | MR | 0.7023 | 1.04 | 0.86–1.25 | 475 | 156 |
| Nm | ER- | AE | 0.999 | 1 | 0.89–1.12 | 937 | 401 |

Table S10. Univariate Cox analysis of the prognostic value of WTAP in breast cancer by clinicopathological factors.

| Event criteria | | | p value | HR | 95% CI | No. patients | No. events |
| --- | --- | --- | --- | --- | --- | --- | --- |
| N- | ER+ | MR | **0.0001** | 0.77 | 0.67–0.88 | 1 389 | 312 |
| N- | ER+ | AE | **0.0005** | 0.82 | 0.73–0.92 | 1 724 | 503 |
| Nm | ER+ | MR | **0.0006** | 0.84 | 0.77–0.93 | 2 757 | 658 |
| Nm | ER+ | AE | **0.0024** | 0.89 | 0.82–0.96 | 3 547 | 1 123 |
| N- | ERm | MR | **0.0033** | 0.85 | 0.76–0.95 | 1 887 | 454 |
| Nm | ERm | MR | **0.0108** | 0.9 | 0.84–0.98 | 3 826 | 993 |
| N- | ERm | AE | **0.0121** | 0.89 | 0.81–0.97 | 2 361 | 719 |
| Nm | ERm | AE | **0.0314** | 0.93 | 0.88–0.99 | 4 953 | 1 653 |
| Nm | ER- | MR | 0.2889 | 0.93 | 0.82–1.06 | 1 039 | 330 |
| N+ | ER+ | MR | 0.2929 | 0.91 | 0.76–1.09 | 677 | 202 |
| Nm | ER- | AE | 0.3701 | 0.95 | 0.86–1.06 | 1 365 | 520 |
| N+ | ER+ | AE | 0.4229 | 0.94 | 0.82–1.09 | 983 | 397 |
| N- | ER- | MR | 0.4418 | 0.93 | 0.77–1.12 | 479 | 140 |
| N- | ER- | AE | 0.4846 | 0.95 | 0.81–1.10 | 612 | 211 |
| N+ | ERm | MR | 0.6141 | 0.96 | 0.84–1.11 | 980 | 322 |
| N+ | ERm | AE | 0.7846 | 0.98 | 0.88–1.10 | 1 418 | 608 |
| N+ | ER- | MR | 0.8494 | 0.98 | 0.79–1.22 | 295 | 119 |
| N+ | ER- | AE | 0.9107 | 0.99 | 0.83–1.18 | 426 | 210 |

Table S11. Univariate Cox analysis of the prognostic value of KIAA1429 in breast cancer by clinicopathological factors.

| Event criteria | | | p value | HR | 95% CI | No. patients | No. events |
| --- | --- | --- | --- | --- | --- | --- | --- |
| N- | **ER+** | **AE** | **0.0027** | **1.35** | **1.11–1.63** | **506** | **153** |
| **N-** | **ERm** | **AE** | **0.0052** | **1.28** | **1.08–1.52** | **683** | **206** |
| N- | ER+ | MR | 0.0824 | 1.43 | 0.95–2.15 | 277 | 47 |
| Nm | ER+ | MR | 0.0834 | 1.18 | 0.98–1.41 | 816 | 167 |
| Nm | ER+ | AE | 0.0969 | 1.1 | 0.98–1.23 | 1 263 | 400 |
| N+ | ERm | AE | 0.176 | 0.9 | 0.78–1.05 | 558 | 235 |
| N+ | ER- | AE | 0.1911 | 0.74 | 0.47–1.16 | 123 | 65 |
| N- | ERm | MR | 0.2516 | 1.23 | 0.86–1.75 | 357 | 63 |
| N+ | ER- | MR | 0.3558 | 0.59 | 0.19–1.80 | 33 | 18 |
| N+ | ER+ | MR | 0.5358 | 1.1 | 0.82–1.47 | 222 | 58 |
| Nm | ERm | MR | 0.5406 | 1.05 | 0.90–1.23 | 1 079 | 245 |
| Nm | ERm | AE | 0.5476 | 1.03 | 0.93–1.14 | 1 720 | 569 |
| Nm | ER- | AE | 0.6404 | 0.95 | 0.76–1.19 | 443 | 167 |
| Nm | ER- | MR | 0.7332 | 0.94 | 0.67–1.32 | 256 | 77 |
| N+ | ER+ | AE | 0.7377 | 0.97 | 0.83–1.15 | 434 | 170 |
| N- | ER- | AE | 0.7469 | 1.07 | 0.72–1.57 | 166 | 51 |
| N- | ER- | MR | 0.8775 | 0.94 | 0.45–1.98 | 73 | 15 |
| N+ | ERm | MR | 0.9073 | 0.98 | 0.75–1.29 | 255 | 76 |

Table S12. Univariate Cox analysis of the prognostic value of FTO in breast cancer by clinicopathological factors.

| Event criteria | | | p value | HR | 95% CI | No. patients | No. events |
| --- | --- | --- | --- | --- | --- | --- | --- |
| Nm | ER+ | MR | **< 0.0001** | 0.81 | 0.75–0.88 | 2 597 | 606 |
| Nm | ERm | MR | **< 0.0001** | 0.85 | 0.79–0.90 | 3 560 | 898 |
| Nm | ER+ | AE | **< 0.0001** | 0.87 | 0.82–0.92 | 3 606 | 1 163 |
| N- | ER+ | MR | **< 0.0001** | 0.74 | 0.65–0.84 | 1 300 | 290 |
| Nm | ERm | AE | **< 0.0001** | 0.9 | 0.86–0.94 | 5 026 | 1 709 |
| N- | ERm | MR | **< 0.0001** | 0.8 | 0.72–0.89 | 1 741 | 417 |
| N- | ER+ | AE | **0.0016** | 0.86 | 0.78–0.94 | 1 635 | 481 |
| N- | ERm | AE | **0.0028** | 0.89 | 0.82–0.96 | 2 215 | 682 |
| N+ | ERm | MR | 0.2804 | 0.94 | 0.83–1.05 | 860 | 264 |
| N+ | ER+ | MR | 0.4209 | 0.94 | 0.82–1.09 | 606 | 172 |
| Nm | ER- | AE | 0.5823 | 1.03 | 0.93–1.13 | 1 371 | 535 |
| N+ | ER- | MR | 0.5913 | 0.94 | 0.75–1.18 | 246 | 91 |
| N+ | ER+ | AE | 0.6292 | 0.98 | 0.88–1.08 | 912 | 367 |
| N- | ER- | AE | 0.6877 | 1.03 | 0.89–1.20 | 555 | 196 |
| N+ | ERm | AE | 0.7346 | 0.99 | 0.91–1.07 | 1 298 | 550 |
| Nm | ER- | MR | 0.7446 | 0.98 | 0.86–1.11 | 933 | 287 |
| N- | ER- | MR | 0.9633 | 1 | 0.82–1.23 | 422 | 125 |
| N+ | ER- | AE | 0.9936 | 1 | 0.85–1.18 | 377 | 182 |

Table S13. Univariate Cox analysis of the prognostic value of ALKBH5 in breast cancer by clinicopathological factors.

| Event criteria | | | p value | HR | 95% CI | No. patients | No. events |
| --- | --- | --- | --- | --- | --- | --- | --- |
| N+ | ER+ | MR | 0.0664 | 1.18 | 0.99–1.40 | 475 | 156 |
| Nm | ERm | MR | 0.1493 | 1.07 | 0.97–1.18 | 1 846 | 501 |
| N- | ER- | MR | 0.1684 | 1.21 | 0.92–1.58 | 205 | 53 |
| Nm | ER+ | MR | 0.1819 | 1.09 | 0.96–1.22 | 1 337 | 333 |
| Nm | ER+ | AE | 0.3185 | 1.04 | 0.96–1.13 | 2 236 | 804 |
| N+ | ERm | MR | 0.3416 | 1.07 | 0.93–1.24 | 612 | 224 |
| N+ | ER- | MR | 0.4054 | 0.89 | 0.68–1.17 | 135 | 68 |
| N+ | ER+ | AE | 0.4447 | 1.05 | 0.93–1.19 | 781 | 344 |
| Nm | ER- | MR | 0.545 | 1.05 | 0.89–1.25 | 490 | 165 |
| N- | ER+ | MR | 0.63 | 0.95 | 0.79–1.15 | 542 | 113 |
| N- | ER- | AE | 0.6407 | 1.05 | 0.86–1.28 | 338 | 115 |
| N- | ERm | MR | 0.6537 | 1.04 | 0.88–1.22 | 762 | 167 |
| Nm | ERm | AE | 0.6869 | 1.01 | 0.95–1.08 | 3 154 | 1 194 |
| N+ | ER- | AE | 0.7827 | 0.97 | 0.81–1.17 | 266 | 153 |
| N+ | ERm | AE | 0.8112 | 1.01 | 0.91–1.12 | 1 050 | 497 |
| N- | ERm | AE | 0.8167 | 0.99 | 0.88–1.10 | 1 236 | 395 |
| Nm | ER- | AE | 0.9022 | 1.01 | 0.90–1.13 | 880 | 382 |
| N- | ER+ | AE | 0.9854 | 1 | 0.87–1.15 | 877 | 276 |
